# Supplementary material for: The Transcription Factor OVOL2 Represses ID2 and Drives Differentiation of Trophoblast Stem Cells and Placental Development in Mice
Source: Cells. 2020 Mar 31;9(4):840. doi: 10.3390/cells9040840 (PMC7226816; doi:10.3390/cells9040840)
Supplement: Supplementary file 1 [file cells-09-00840-s001.pdf]

Supplementary Materials

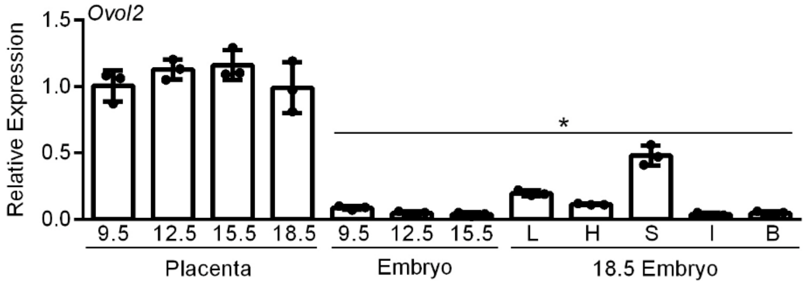

Figure S1. Quantitative analysis of *Ovol2* expression in mouse placenta and embryo throughout gestation. Quantitative RT-PCR analysis showing expression of *Ovol2* in mouse placenta and embryo on E9.5, 12.5, and 15.5. *Ovol2* expression in placenta and embryonic liver (L), heart (H), skin (S), intestine (I), and brain (B) were also assessed on E18.5. Values significantly different from E9.5 placenta (N=3, P<0.05) are indicated with an asterisk (*). Graphs represent means (SD).

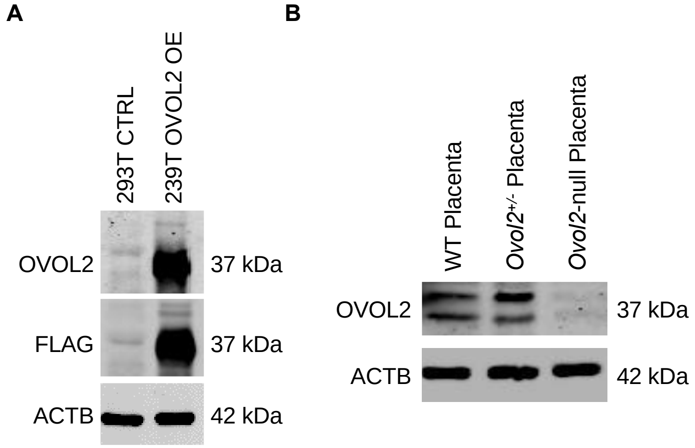

Figure S2. Verification of OVOL2 antibody. Western blot showing OVOL2 expression in (**A**) HEK-293T cells transfected with either pCMV-empty (293T CTRL) or pCMV-OVOL2-FLAG (293T OVOL2 OE). (**B**) Western blot showing OVOL2 expression in wild-type (WT), *Ovol2*-heterozygote (*Ovol2*$^{+/-}$), or *Ovol2*-null placentas. Molecular weight of OVOL2 (A isoform) is 37 kDa. ACTB was used as a loading control.

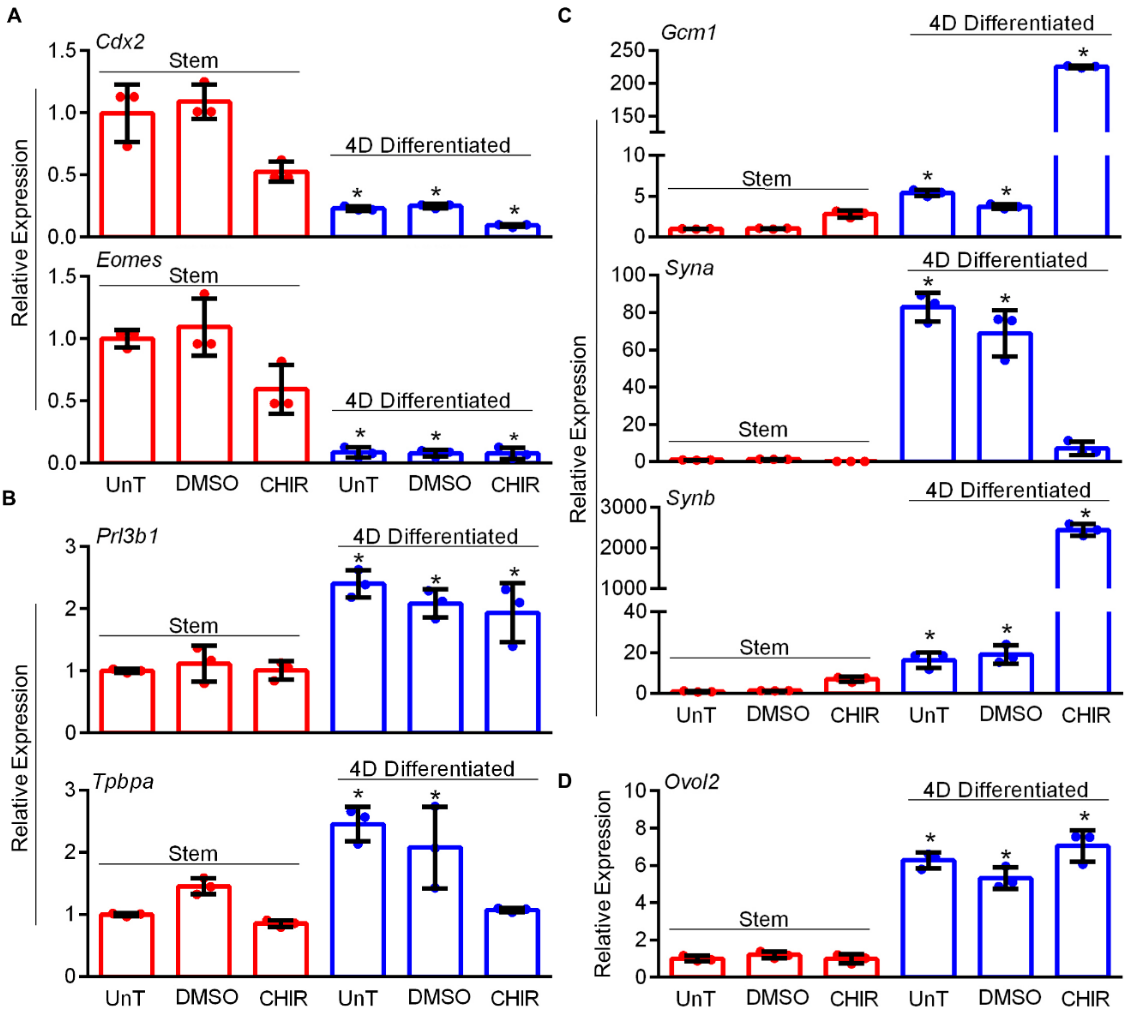

Figure S3. Transcript analysis of mouse TS cells cultured under stem conditions versus two differentiation protocols. Quantitative RT-PCR analysis of (**A**) stem (*Cdx2* and *Eomes*), (**B**) junctional zone-associated transcripts (*Prl3b1* and *Tpbpa*), (**C**) labyrinth zone-associated transcripts (*Gcm1*, *Syna*, *Synb*), and (**D**) *Ovol2* following culture of TS cells in stem conditions or four days of differentiation conditions. In both stem and differentiation conditions, cells were untreated (UnT), exposed to DMSO (vehicle), or exposed to the GSK3β inhibitor CHIR99021 (CHIR). Note that TS cells exposed to differentiation conditions in the presence of CHIR preferentially differentiated to the syncytiotrophoblast II lineage. *Ovol2* expression increased in both differentiation protocols. Values significantly different from stem, UnT conditions ($N = 3$, $P < 0.05$) are indicated with an asterisk (*). Graphs represent means (SD).
